# Author Correction: Photothermal effects control ultrafast charge transport in titanium carbide MXenes

**DOI:** 10.1038/s41467-026-75343-8

**Published:** 2026-07-09

**Authors:** Wenhao Zheng, Hugh Ramsden, Stefano Ippolito, Max van Hemert, Danzhen Zhang, Teng Zhang, Dongqi Li, Guanzhao Wen, Jaco J. Geuchies, Minghao Yu, Xinliang Feng, Yury Gogotsi, Klaas-Jan Tielrooij, Hai I. Wang

**Affiliations:** 1 Max Planck Institute for Polymer Research, Ackermannweg 10, Mainz, Germany; 2 Department of Physics, Massachusetts Institute of Technology, Cambridge, MA USA; 3 GBA Branch of Aerospace Information Research Institute, Chinese Academy of Sciences, Guangzhou, China; 4 Department of Applied Physics, TU Eindhoven 5612, AZ Den Dolech 2, Eindhoven, the Netherlands; 5 A. J. Drexel Nanomaterials Institute, Department of Materials Science and Engineering, Drexel University, 3141 Chestnut St, Philadelphia, PA USA; 6 Center for Advancing Electronics Dresden (CFAED) & Faculty of Chemistry and Food Chemistry, Technische Universität Dresden, Mommsenstrasse 4, Dresden, Germany; 7 Leiden Institute of Chemistry, Leiden University, Einsteinweg 55, Leiden, the Netherlands; 8 Max Planck Institute of Microstructure Physics, Weinberg 2, Halle, Germany; 9 Catalan Institute of Nanoscience and Nanotechnology (ICN2), BIST and CSIC, Campus UAB, Bellaterra (Barcelona), 08193 Spain; 10 Nanophotonics, Debye Institute for Nanomaterials Science, Utrecht University, Utrecht, the Netherlands

**Keywords:** Two-dimensional materials, Condensed-matter physics, Optical techniques

Correction to: *Nature Communications*10.1038/s41467-026-68831-4, published online 29 January 2026

In the version of this article originally published, an error was introduced during the preparation of Fig. 3e. The calculated photoconductivity map was displayed using an inappropriate colourscale limit, which caused values below the lower limit to be clipped and represented by the same saturated colour. Consequently, the colour representation in part of Fig. 3e did not accurately reflect the full calculated variation in Δσ. Fig. 3e has now been corrected in the HTML and PDF versions of the article, as seen below.

**Original Fig. 3e** 
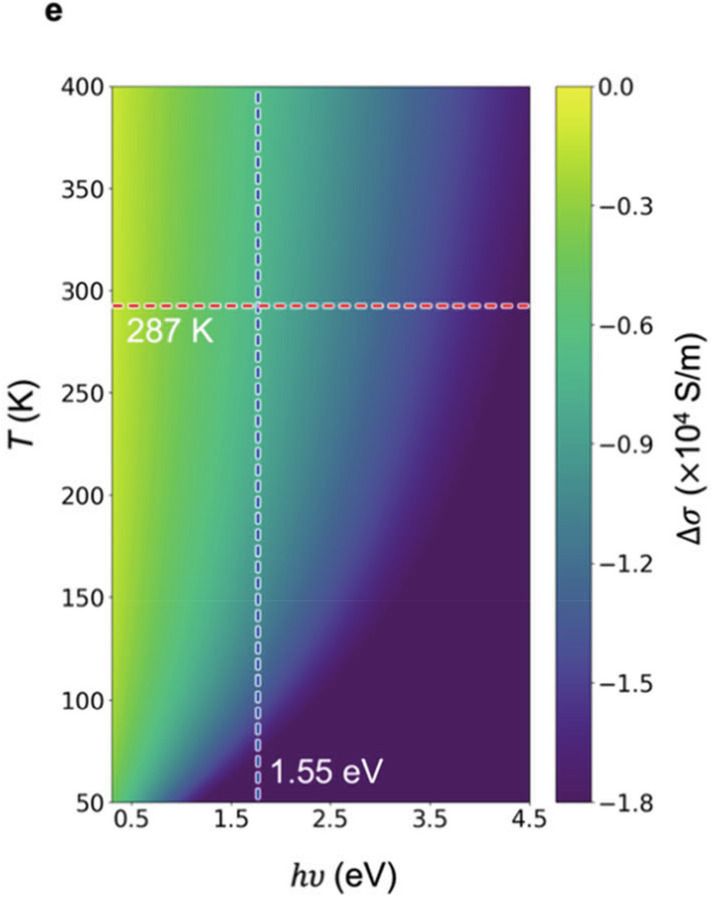


**Corrected Fig. 3e** 
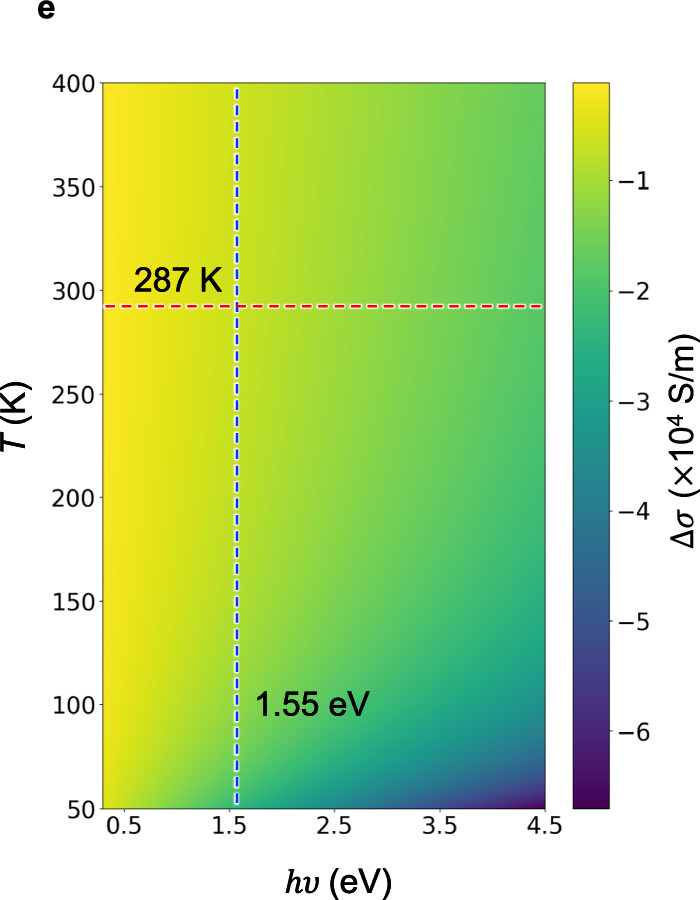


The underlying simulation data and calculations were not affected. The vertical and horizontal cuts used for comparison with the experimental data in Fig. 3b and d remain unchanged. This correction does not affect the quantitative analysis, interpretation, or conclusions of the article.

