# Supplementary material for: Photothermal effects control ultrafast charge transport in titanium carbide MXenes
Source: Nat Commun. 2026 Jan 29;17:1201. doi: 10.1038/s41467-026-68831-4 (PMC12858860; doi:10.1038/s41467-026-68831-4)
Supplement: Supplementary file 1 — Supplementary Information [file 41467_2026_68831_MOESM1_ESM.pdf]

Supplementary Information for:

Photothermal effects control ultrafast charge transport in titanium carbide MXenes

Wenhao Zheng[1, 2, 3], Hugh Ramsden[4], Stefano Ippolito[5], Max van Hemert[4], Danzhen Zhang[5], Teng Zhang[5], Dongqi Li[6], Guanzhao Wen[1], Jaco J. Geuchies[1,8], Minghao Yu[6, 7], Xinliang Feng[6, 7], Yury Gogotsi[5], Klaas-Jan Tielrooij[4,9], Hai I. Wang[*1, 10]

[1] Max Planck Institute for Polymer Research, Ackermannweg 10, 55128 Mainz, Germany;

[2] Department of Physics, Massachusetts Institute of Technology, Cambridge, MA, USA;

[3] GBA Branch of Aerospace Information Research Institute, Chinese Academy of Sciences, Guangzhou 510700, China

[4] Department of Applied Physics, TU Eindhoven, 5612 AZ Eindhoven, the Netherlands;

[5] A. J. Drexel Nanomaterials Institute, Department of Materials Science and Engineering, Drexel University, 3141 Chestnut St, Philadelphia, PA 19104, USA;

[6] Center for Advancing Electronics Dresden (CFAED) & Faculty of Chemistry and Food Chemistry, Technische Universität Dresden, Mommsenstrasse 4, 01062 Dresden, Germany;

[7] Max Planck Institute of Microstructure Physics, Weinberg 2, 06120 Halle, Germany;

[8] Leiden Institute of Chemistry, Leiden University, Einsteinweg 55, 2333CC, Leiden, the Netherlands;

[9] Catalan Institute of Nanoscience and Nanotechnology - ICN2 (BIST and CSIC), Campus UAB, Bellaterra, Barcelona, Spain

[10] Nanophotonics, Debye Institute for Nanomaterials Science, Utrecht University, The Netherlands.

*Email: h.wang5@uu.nl

Table of Contents

Supplementary section 1. Terahertz optical response of $Ti_3C_2T_x$ thin film (page 3);

Supplementary section 2. Temperature dependence of ω_p^2 in $Ti_3C_2T_x$ thin film (page 4);

Supplementary section 3. Photon-energy dependence of the maximum photoconductivity (page 4);

Supplementary section 4. Photon-energy dependence of the averaged photoconductivity (page 5);

Supplementary section 5. Comparison of photoconductivity dynamics under 400 and 800 nm pump (page 5);

Supplementary section 6. Logic flow for the photoconductivity modeling (page 6);

Supplementary section 7. Details of transient reflectivity measurements (page 6).

Supplementary section 8. Power dependence of transient reflectivity (page 7).

Section 1. Terahertz optical response of $Ti_3C_2T_X$ thin film

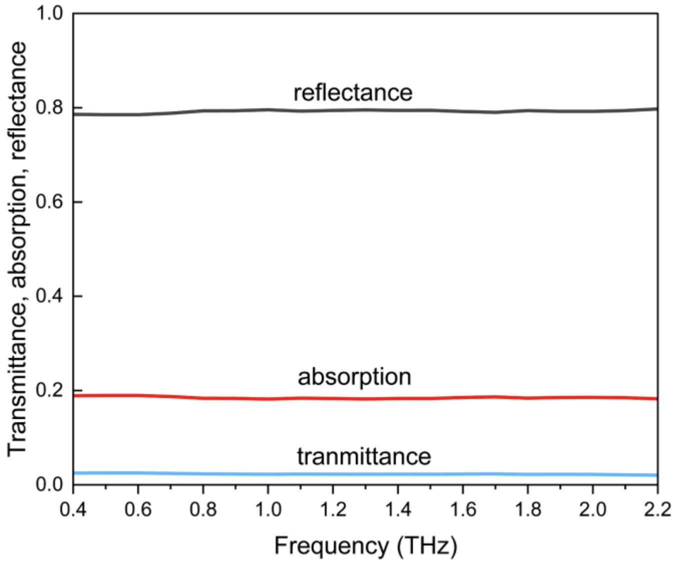

Supplementary Figure 1. Experimental THz optical response of a ~25 nm $Ti_3C_2T_x$ film on a quartz substrate. The reflectance R (black) is ~79.1% across 0.5–2.2 THz, while the transmittance T (blue) is ~2.4 % and the true absorptance A (red), defined as $A = 1 - R - T$, is ~18.5%.

Transmittance (T), reflectance (R), and absorptance (A) are calculated using the standard sheet-impedance model for a conductive thin film on a dielectric substrate (refractive index $n_s = 1.96$). For a film much thinner than the THz wavelength (with no need to include multiple internal reflections inside the film), with sheet resistance $R_s = \frac{1}{\sigma}$), the amplitude Fresnel coefficients at normal incidence read (air → film → substrate):

$$r = \frac{n_s - 1 - Z_0/R_s}{n_s + 1 + Z_0/R_s}, \qquad t = \frac{2}{1 + n_s + Z_0/R_s}, \qquad R = |r|^2, \qquad T = n_s|t|^2, \qquad A = 1 - R - T$$

where Z_0 is the wave impedance in vacuum, σ is the complex conductivity, n_0 is the refractive index of air, n_s is the refractive index of the substrate, and d is the thickness of the sample. This yields the total reflectance and transmittance of the sample-substrate system.

Using the impedance-matching thin-film model with our experimentally determined average conductivity (1.6×10^6 S/m) and film thickness (~25 nm), we obtain a theoretical absorptance of ~18.5%, which is in excellent agreement with the experimentally measured value.[1]

Section 2. Temperature dependence of ω_p^2 in $Ti_3C_2T_X$ thin film

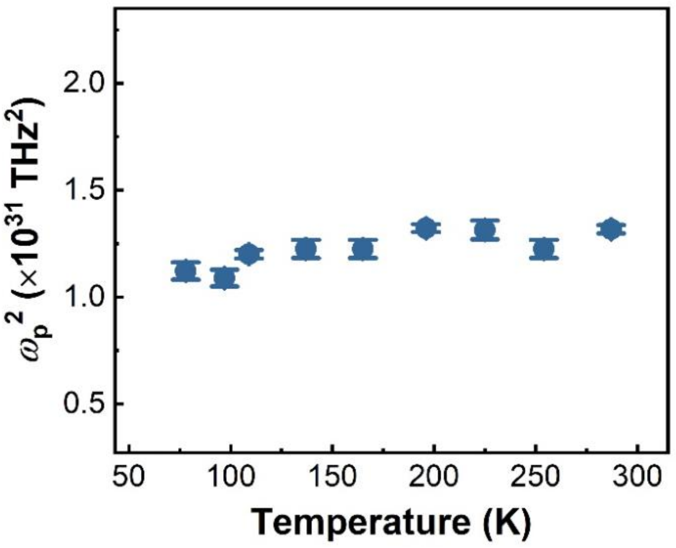

Supplementary Figure 2. **Temperature dependence of ω_p^2 in $Ti_3C_2T_x$ thin film.** Temperature-dependent squared plasma frequency (ω_p^2), which is proportional to the carrier density, measured for $Ti_3C_2T_x$ thin film. Error bars represent the standard errors of ω_p^2 obtained from Drude model fitting to the complex terahertz conductivity spectra at each temperature.

Section 3. Photon-energy dependence of the maximum photoconductivity

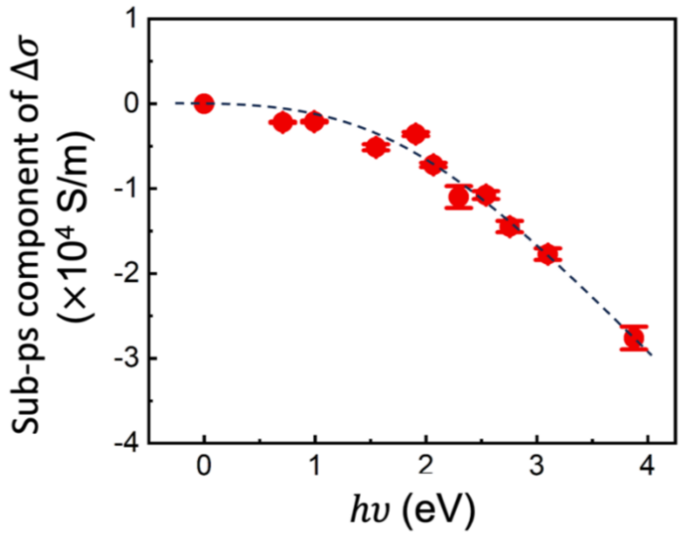

Supplementary Figure 3. **Photon-energy dependence of the maximum photoconductivity**. The photon energy-dependent sub-picosecond components, which is obtained by subtracting the average values between 2-5 ps from the peak photoconductivity magnitudes. Error bars

represent the standard deviation of the photoconductivity values averaged over the 2–5 ps time window.

Section 4. Photon-energy dependence of the averaged photoconductivity.

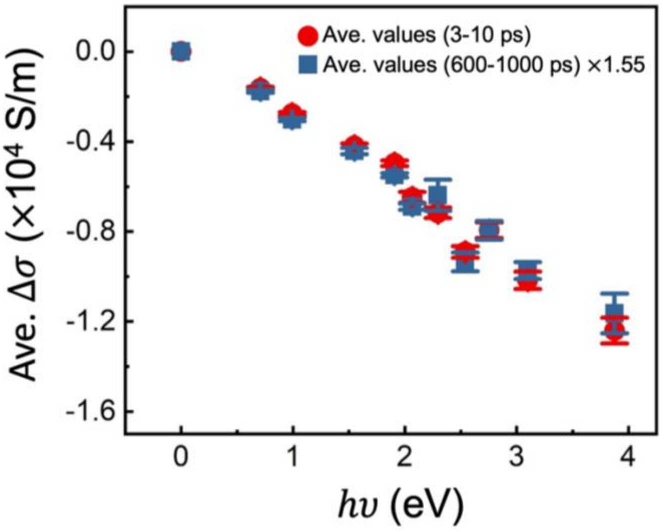

Supplementary Figure 4. **Photon-energy dependence of the averaged photoconductivity.** Photoconductivity values averaged over 2–10 ps (red circles) and 600–1000 ps (blue squares) as a function of photon energy. The 600–1000 ps data are scaled by a factor of 1.55 for direct comparison. Error bars represent the standard deviation of the photoconductivity values averaged over the respective time windows (2–10 ps and 600–1000 ps).

Section 5. Comparison of photoconductivity dynamics under 400 and 800 nm pump

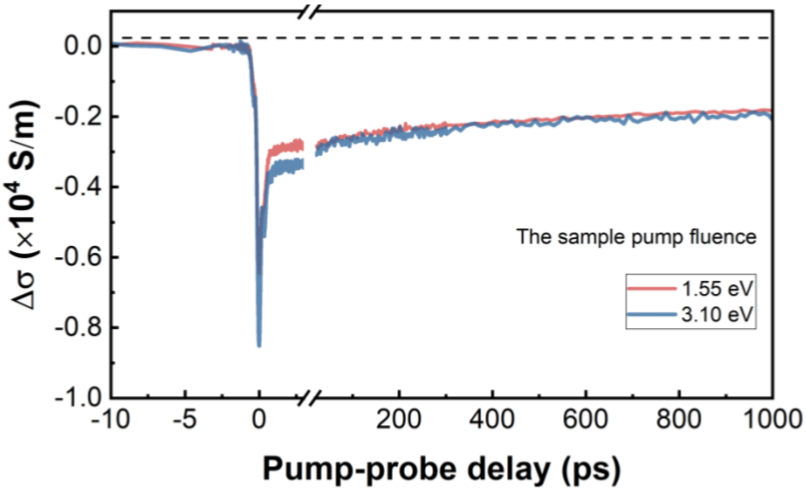

Supplementary Figure 5. **Comparison of photoconductivity dynamics under different pump photon energies**. Time-resolved photoconductivity change ($\Delta\sigma$) of $Ti_3C_2T_x$ MXene

under optical excitation at two photon energies: 1.55 eV (red) and 3.10 eV (blue), with the same pump fluence.

Section 6. Logic flow for the photoconductivity modeling

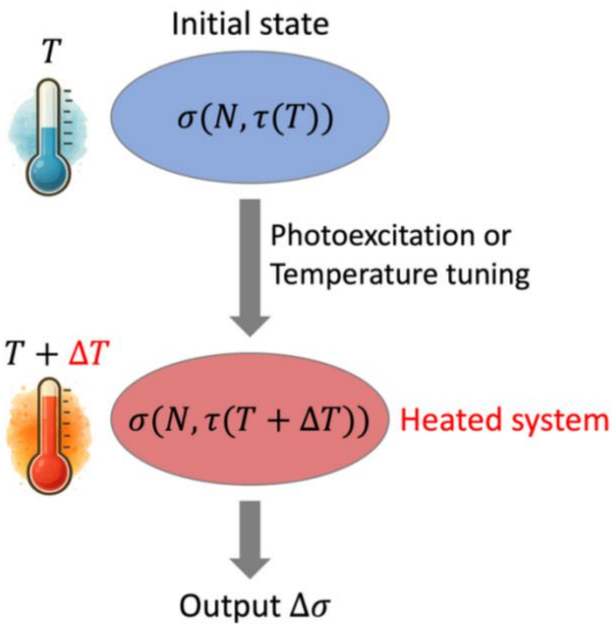

Supplementary Figure 6. Schematic illustrating the microscopic model for calculating photoconductivity changes ($\Delta\sigma$) based on modulating the microscopic transport parameters (e.g. momentum scattering time τ) following light absorption and/or tuning T.

Section 7. Details of transient reflectivity measurements

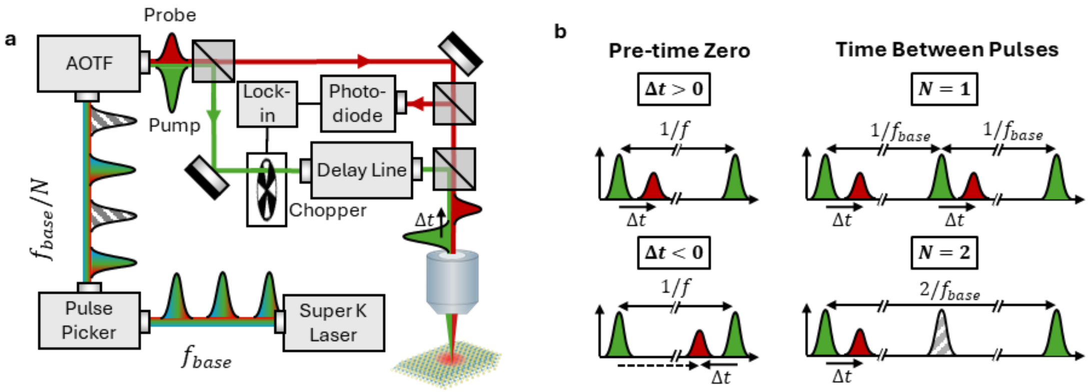

Supplementary Figure 7. Transient reflectivity measurement details. a, Schematic diagram of transient reflectivity setup. Pulsed white light is generated by a supercontinuum laser with a

repetition rate f_{base}. This decrease to $\mathrm{f_{base}}/N$ using a pulse picker, where N is an integer. The pump and probe wavelengths are selected using an acousto-optic tuneable filter (AOTF). A delay stage is used to delay the pump allowing the probe to arrive at the sample offset in time by $\Delta\mathrm{t}$. $\Delta\mathrm{R}/\mathrm{R}$ is measured through the use of a photodiode and lock-in amplification. **b,** Illustration of pre-time zero and time between pulses. For positive delays, $\Delta t > 0$, the probe arrives after the pump. For negative probe delays, the probe arrives $1/\mathrm{f} + \Delta t$ after the previous pump. If $\Delta t < 0$ and small, we can define an 'effective' delay $\Delta t_{eff} \approx 1/f = N/f_{base}$. **c,** The time between pulses is given by N/f_{base}. For larger values of N, we extend this window, increasing the time between pulses.

Section 8. Power dependence of transient reflectivity.

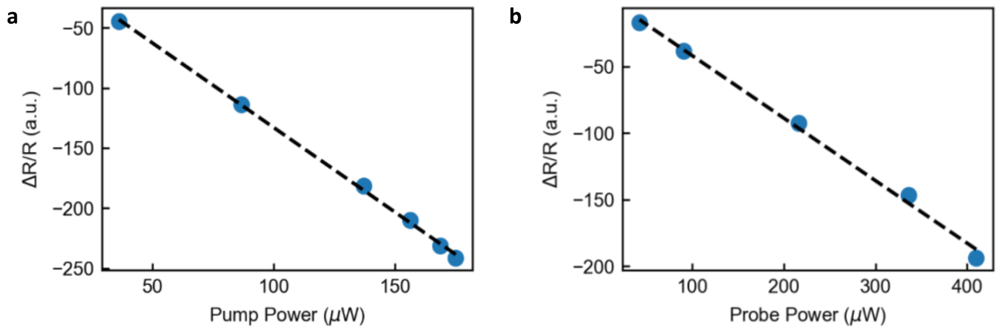

Supplementary Figure 8. Power dependence of transient reflectivity. **a and b,** Plot of measured transient reflectivity vs average pump (a) and probe (b) power in the regimes used in these measurements. Linear fits are shown in blacked dashed lines. For the pump scan, the probe power was fixed at 137 μW, for the probe scan, the pump was fixed at 175 μW. Assuming linear absorption of the pump, we expect the temperature increase from the pump to be linear with power, as is demonstrated in (a). In (b) we demonstrate that we are operating in the linear regime for the probe.

Supplementary references

[1] Zhao, T. *et al.* Ultrathin MXene assemblies approach the intrinsic absorption limit in the 0.5–10 THz band. *Nat. Photonics* **17**, 622-628, (2023).
